# Hospital Length of Stay After Hip Fracture and It’s Association With 4-Month Mortality—Exploring the Role of Patient Characteristics

**DOI:** 10.1093/gerona/glab302

**Published:** 2021-10-08

**Authors:** Stina Ek, Anna C Meyer, Margareta Hedström, Karin Modig

**Affiliations:** Unit of Epidemiology, Institute of Environmental Medicine, Karolinska Institutet, Stockholm, Sweden; Unit of Epidemiology, Institute of Environmental Medicine, Karolinska Institutet, Stockholm, Sweden; Department of Orthopedics, Karolinska University Hospital, Stockholm, Sweden; Department of Clinical Science, Intervention and Technology, Karolinska Institutet, Stockholm, Sweden; Unit of Epidemiology, Institute of Environmental Medicine, Karolinska Institutet, Stockholm, Sweden

**Keywords:** Health care, Injury, National registers, Prognosis

## Abstract

**Background:**

Hospital length of stay (LoS) is believed to be associated with higher mortality in hip fracture patients; however, previous research has shown conflicting results. We aimed to explore the association between LoS and 4-month mortality in different groups of hip fracture patients.

**Methods:**

The study population in this Swedish register-based cohort study was 47 811 patients 65 years or older with a first hip fracture during 2012–2016, followed up for 4 months after discharge. LoS was categorized by cubic splines, and the association between LoS and mortality was analyzed with Cox regression models, adjusted for sociodemographic- and health-related factors.

**Results:**

Mean LoS was 11.2 ± 5.9 days and 12.3% of the patients died within 4 months. Both a shorter and a longer LoS, compared to the reference 9–12 days, were associated with higher mortality (hazard ratio [95% confidence interval]): 2–4 days 2.15 (1.98–2.34), 5–8 days 1.58 (1.47–1.69), and 24+ days 1.29 (1.13–1.46). However, in fully adjusted models, only the association with a long LoS remained: 13–23 days 1.08 (1.00–1.17) and 24+ days 1.42 (1.25–1.61). Stratifying by living arrangement revealed that the increased risk for a short LoS was driven by the group living in care homes. For patients living at home, a short LoS was associated with lower risk: 0.65 (0.47–0.91) and 0.85 (0.74–0.98) for 2–4 and 5–8 days, respectively.

**Conclusions:**

A long LoS after a hip fracture is associated with increased 4-month mortality risk even after considering patient characteristics. The association between mortality and a short LoS, however, is explained by individuals coming from care homes (with higher mortality risk), being discharged early.

The lifetime risk of hip fractures is approximately 10% for men and 20% for women, and hip fractures often lead to long hospitalizations, dependence, physical disability, lower quality of life, and an earlier death (1–4). Many factors influence the prognosis and death risk of hip fracture patients. One factor that has changed quite drastically over time in Sweden, but has not been explored in depth, is the time patients stay at the hospital after their hip fracture. Several studies have shown a trend toward the shorter length of stay (LoS) over time (5–7). When policies change and fewer hospital beds are available, there might be a risk for discharging patients too early with negative health outcomes as a consequence. On the contrary, an early discharge is associated with an earlier mobilization of patients that is related to a better prognosis (8). In an ideal setting, the LoS is based on every individual’s own needs and prerequisites; however, other factors may influence the LoS as well, such as shortage of hospital beds or a desire from the patient to go home.

A study from the United States shows that a short LoS is associated with a lower mortality rate among hip fracture patients (9). Other studies contradict these findings, showing that a short LoS is associated with higher mortality (10,11). In a Swedish study from 2015, Nordström et al. (7) show that the association between LoS and mortality might not be linear but rather U- or J-shaped, which could explain some of the scattered results from previous studies. The LoS is likely related to the health status of the patient, in terms of healthier patients being discharged earlier and those with poorer health later (12,13), potentially explaining why longer hospital stays have been related to higher mortality. The study from Nordström et al. did not have the possibility to handle the crucial aspect of possible confounding by characteristics of the patients, such as health status or sociodemographic factors.

Comparing studies from different countries and regions might be problematic because health care systems and policies differ across countries and regions. Such differences are likely to affect LoS. For example, some health care systems might rely on posthospital rehabilitation units while other systems keep the patient at the hospital until rehabilitated, and this would have a large impact on the LoS. According to Swedish guidelines, 80% of all hip fractures are to receive surgery within 24 hours from admission, and almost all hip fracture patients have received surgery within 48 hours. Furthermore, all patients shall be mobilized the day after surgery and before they are being admitted elsewhere. If there is a need for long-term rehabilitation, the patient is normally referred to short-term care homes with that specific purpose (14). Despite regional differences, it is important to study if LoS affects mortality because it is an exposure that is modifiable with health care guidelines and updated policies.

With this study, we aim to investigate the relationship between LoS and 4-month mortality after a hip fracture, taking into consideration patients’ health status, physical function, and sociodemographic factors.

## Method

### Data

Individuals in Sweden aged 65 years and older admitted to the hospital with an incident hip fracture between the years 2012 and 2016 were identified in the Swedish National Patient Register (NPR), using ICD-10 codes S720–S722. Information about the outcome death within 4 months was extracted from the Cause of Death Register, and information about sociodemographic factors and health status was extracted from the Swedish Hip Fracture Register (SHR). The different sources were linked to each other with the Swedish Personal Number (PIN) assigned to all individuals residing in Sweden. The PIN was replaced with an identification number in the database and consequently anonymized. The NPR and the Cause of Death Register are administrative registers with close to full national coverage, while the SHR is a clinical register with a coverage of 80%–90% of all hip fractures in Sweden during the study period (15).

In total, 60 382 hip fractures were identified in both NPR and SHR. We excluded patients who did not receive surgery or who had a pathological fracture (*n* = 123 and *n* = 703) due to expected differences in health care utilization, as well as individuals with an outlier number of days spent at the hospital (LoS <2 *n* = 362, LoS >30 *n* = 2 655). We also excluded those who died during the hospital stay (*n* = 2 697), because the follow-up started at discharge from the hospital. Because LoS was defined as a stay at the index hospitalization, we also excluded individuals who were transferred to another hospital or ward (*n* = 3 456). Last, individuals with missing values in any of the included variables were excluded (*n* = 2 575). The final analytical sample consisted of 47 811 individuals aged 65 years or older who had endured their first hip fracture anytime during the period 2012–2016.

The exposure LoS was based on data from the NPR and was calculated in days, spanning from the day that the patient was admitted to a hospital due to a hip fracture until the day the patient was discharged.

#### Covariates

Age and sex were gathered from the NPR. American Society of Anesthesiologists physical status classification (ASA score) (16), walking ability, and living arrangements before admission were retrieved from the SHR. ASA score spans from 1 to 6, where 1 translates into being healthy and 6 is deceased and was in this study categorized into 1/2/3/4 + 5. Walking ability was self-reported and categorized into independent/assisted outside/independent inside/assisted inside/not able to walk. The type of facility that the patient had come from (living arrangements before admission) was categorized into own home/care home or other type of service facility/other health care facility (other hospital or another ward). Type of hip fracture was categorized into intracapsular/pertrochanteric/subtrochanteric.

### Statistical Analysis

Descriptive information about the study population was stratified by sex and presented as percentages or means. To test and visualize the shape of the association, we first introduced a restricted cubic spline with 4 knots and then used the postestimation command “xlbs” (17) to obtain hazard ratios (HRs; 95% CI) for all different values of LOS (2–30) using the mean LoS as a reference (11.2 ± 5.9) to create a graph of the association for each possible LOS. After concluding the nonlinearity of the association, we created a new categorical variable of LoS based on the restricted cubic splines (2–4, 5–8, 9–12, 13–23, and 24–30 days). Absolute risks of death within 4 months for the different categories of LoS were calculated, for all and stratified by sex and age groups. The risk of death over time was analyzed with Cox proportional hazards models, with the categories of LoS as exposure, using the category including the mean LoS for this population (9–12 days) as a reference and controlling for age and sex. To explore the impact of other sociodemographic and health-related factors, we adjusted for ASA score, walking ability, and living arrangements before admission, one by one and in a final model including all of them. Last, an interaction term between LoS and living arrangements was introduced, and consequently, we also stratified by living arrangements before admission, to compare the risk association between independent living and institutionalized/hospitalized older adults.

### Sensitivity Analyses

The Cox proportional hazards models were also performed stratified by sex and age groups, to check for subgroup differences. Finally, we compared death rates within 30 days from admission between those who died outside of the hospital and those who died during the hospital stay, to explore whether the place of death during those first 30 days influenced the death rates.

Statistical analyzes were performed with Stata 16 (StataCorp., College Station, TX).

## Results

Descriptive statistics of the study population are presented in Table 1. The study population consisted of nearly 70% women, and the mean age was 83.0 (±7.9) years. Women were slightly older than men (83.6 ± 7.8 compared to 81.7 ± 8.0). During the 4-month follow-up period, 5 879 (12.3%) individuals died, and men had a higher mortality than women with 15.5% compared to 10.9% dead. The mean LoS was just over 11 days (11.2 ± 5.9). Table 1 also displays causes of death where cardiovascular diseases were the most common. Psychological diseases and injuries/trauma were more common causes of death among individuals with shorter LoS, while respiratory diseases and tumors were more common causes of death among those with long LoS.

**Table 1.** Baseline Characteristics of the Study Population by LoS

	All (*n* = 47 811)	LoS 2–4 days (*n* = 5 114)	LoS 5–8 days (*n* = 12 778)	LoS 9–12 days (*n* = 13 296)	LoS 13–23 days (*n* = 14 470)	LoS 24+ days (*n* = 2 153)
Age, mean (*SD*)	83.0 (7.9)	81.6 (8.8)	82.4 (8.3)	83.1 (7.6)	83.9 (7.4)	84.0 (7.3)
ASA score, *n* (%)						
1	2 257 (4.7)	404 (7.9)	793 (6.2)	604 (4.5)	414 (2.9)	42 (2.0)
2	18 079 (37.8)	1 928 (37.7)	5 010 (39.2)	5 412 (40.7)	5 076 (35.1)	653 (30.3)
3	24 567 (51.4)	2 443 (47.8)	6 272 (49.1)	6 592 (49.6)	8 004 (55.3)	1 256 (58.3)
4–5	2 908 (6.1)	339 (6.6)	688 (5.2)	688 (5.2)	976 (6.7)	202 (9.4)
Walking ability before admission, *n* (%)						
Independent	28 951 (60.6)	2 091 (40.9)	6 735 (52.7)	8 993 (67.6)	9 718 (67.2)	1 414 (65.7)
Assisted outside	4 216 (8.8)	553 (10.8)	1 146 (9.0)	1 021 (7.7)	1 301 (9.0)	195 (9.1)
Independent inside	10 308 (21.6)	1 559 (30.5)	3 247 (25.4)	2 422 (18.2)	2 675 (18.5)	405 (18.8)
Assisted inside	3 100 (6.5)	617 (12.1)	1 204 (9.4)	624 (4.7)	553 (3.8)	102 (4.7)
Not able to walk	1 236 (2.6)	294 (5.8)	446 (3.5)	236 (1.8)	223 (1.5)	37 (1.7)
Living arrangements before admission, *n* (%)						
Own home	34 290 (71.7)	1 786 (34.9)	6 960 (54.5)	10 953 (82.4)	12 695 (87.7)	1 896 (88.1)
Care home or similar	11 617 (24.3)	3 149 (61.6)	5 385 (42.1)	1 856 (14.0)	1 123 (7.8)	104 (4.8)
Other health care facility	1 904 (4.0)	179 (3.5)	433 (3.4)	487 (3.7)	652 (4.5)	153 (7.1)
4-month mortality, *n* (%)	5 879 (12.3)	939 (18.4)	1 842 (14.4)	1 313 (9.9)	1 495 (10.3)	290 (12.3)
Cause of death, *n* (%)*						
Infection	128 (2.2)	16 (1.7)	33 (1.8)	31 (2.4)	37 (2.5)	11 (3.8)
Tumors	706 (12.0)	66 (7.0)	168 (9.1)	188 (14.3)	241 (16.1)	43 (14.8)
Endocrine	110 (1.9)	21 (2.2)	25 (1.4)	30 (2.3)	27 (1.8)	7 (2.4)
Psychological including dementia	811 (13.8)	208 (22.2)	343 (18.6)	127 (9.7)	112 (7.5)	21 (7.2)
Neurological	386 (6.6)	107 (11.4)	161 (8.7)	56 (2.3)	55 (3.7)	7 (2.4)
Cardiovascular	2 022 (34.4)	257 (27.4)	605 (32.8)	475 (36.2)	575 (38.5)	110 (37.9)
Respiratory	246 (4.2)	10 (1.1)	49 (2.7)	67 (5.1)	96 (6.4)	24 (8.3)
Injuries and trauma	1 005 (17.1)	188 (20.0)	343 (18.6)	225 (17.1)	215 (14.4)	34 (11.7)
Other or UNS	465 (7.9)	66 (7.0)	115 (6.2)	114 (8.7)	137 (9.2)	33 (11.4)

*Note:* LoS = length of hospital stay; SD = standard deviation; ASA = American Society of Anesthesiologists physical status classification; UNS = unspecified.

*Subsample of deceased within 4 months, *n* = 5 879.

**Figure 1.**
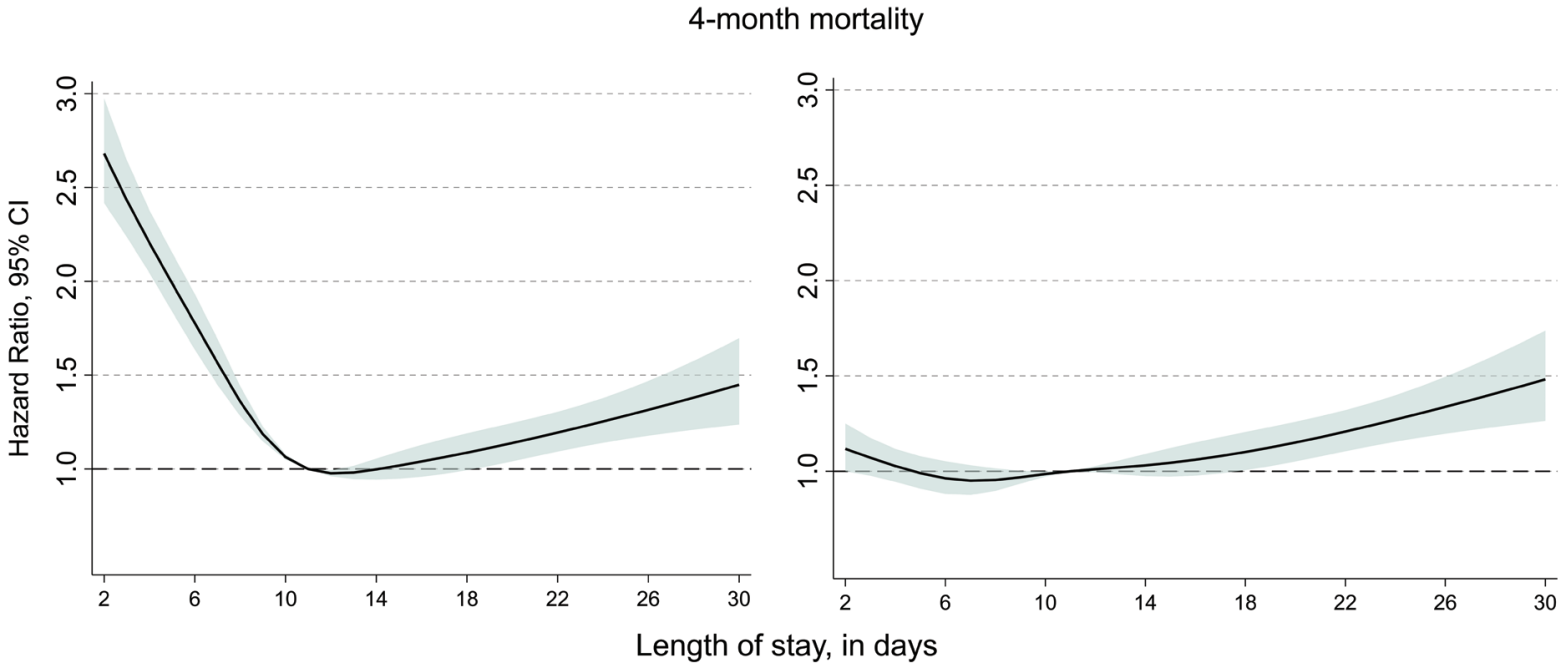
Hazard ratios for the association between days of LoS and 4-month mortality with 11 days as reference: (**A**) unadjusted model and (**B**) fully adjusted model (sex, age, ASA grade, walking ability, and living arrangements). LoS = length of hospital stay; ASA = American Society of Anesthesiologists physical status classification.

Table 2 displays the association between LoS and 4-month mortality estimated by Cox regression. An elevated risk of mortality was found for shorter LoS periods compared to the reference group (LoS 9–12 days): the age- and sex-adjusted HRs for LoS 2–4 days were 2.15 (95% CI: 1.98–2.34) and 1.58 for LoS 5–8 days (95% CI: 1.47–1.69). The longest LoS (≥24 days) was also associated with a slightly increased mortality (HR = 1.29, 95% CI: 1.13–1.46). These results remained similar when separately controlling for ASA score or walking ability. When controlling for type of living arrangement before the admission, only the association with longer LoS remained significant; LoS 13–23 days (HR = 1.14, 95% CI: 1.06–1.23) and LoS 24 days or more (HR = 1.56, 95% CI: 1.37–1.77). In the fully adjusted model, the magnitude and strength of the HRs remained very similar as in the model adjusting only for the type of living arrangement before admission (Table 2). Table 2 also displays that all factors controlled for in the fully adjusted model (apart from age and sex; ASA score, walking ability, and living arrangements before admission) were significantly associated with 4-month mortality independently of each other. The nonlinear relationship between LoS and mortality in the 5 different models is displayed in Figure 1, showing that the nonlinearity in the unadjusted model was flattening when adjusting for ASA grade, walking ability and living arrangement.

**Table 2.** Hazard Ratios for the Association Between LoS and 4-Month Mortality

			Hazard Ratios, 95% CI				
	n	Cases	Model 1	Model 2	Model 3	Model 4	Model 5
LoS, in days							
2–4	5 114	939	**2.15 (1.98–2.34)**	**2.03 (1.87–2.21)**	**1.51 (1.39–1.65)**	1.04 (0.96–1.14)	1.06 (0.97–1.16)
5–8	12 778	1 842	**1.58 (1.47–1.69)**	**1.53 (1.43–1.65)**	**1.27 (1.18–1.36)**	0.98 (0.91–1.15)	0.99 (0.92–1.07)
9–12	13 296	1 313	Ref.	Ref.	Ref.	Ref.	Ref.
13–23	14 470	1 495	0.98 (0.91–1.05)	0.93 (0.86–0.99)	1.01 (0.94–1.09)	**1.14 (1.06–1.23)**	**1.08 (1.00–1.17)**
24+	2 153	290	**1.29 (1.13–1.46)**	**1.17 (1.03–1.33)**	**1.31 (1.15–1.48)**	**1.56 (1.37–1.77)**	**1.42 (1.25–1.61)**
ASA score							
1				Ref.			Ref.
2			—	**1.82 (1.42–2.34)**	—	—	**1.38 (1.08–1.77)**
3			—	**3.68 (2.88–4.69)**	—	—	**2.22 (1.73–2.83)**
4–5			—	**7.27 (5.65–9.35)**	—	—	**3.89 (3.02–5.01)**
Walking ability							
Independent					Ref.		Ref.
Assisted outside			—	—	**2.52 (2.31–2.74)**	—	**1.69 (1.54–1.85)**
Independently inside			—	—	**2.90 (2.72–3.10)**	—	**1.81 (1.69–1.96)**
Assisted inside			—	—	**3.59 (3.29–3.91)**	—	**1.99 (1.81–2.18)**
Not able to walk			—	—	**3.53 (3.12–4.01)**	—	**1.97 (1.73–2.25)**
Living arrangement before admission							
Own living						Ref.	Ref.
Any service accommodation			—	—	—	**3.78 (3.54–4.03)**	**2.34 (2.17–2.52)**
Other health care instance			—	—	—	**2.78 (2.49–3.10)**	**1.89 (1.69–2.11)**

*Notes:* LoS = length of hospital stay; CI = confidence interval; ASA = American Society of Anesthesiologists physical status classification. Model 1: adjusted for age and sex; Model 2: adjusted for age, sex, and ASA score; Model 3: adjusted age, sex, and walking ability before the fracture; Model 4: adjusted for age, sex, and living arrangements before admission; Model 5: adjusted for all factors above. Statistically significant hazard ratios are denoted in bold.

**Figure 2.**
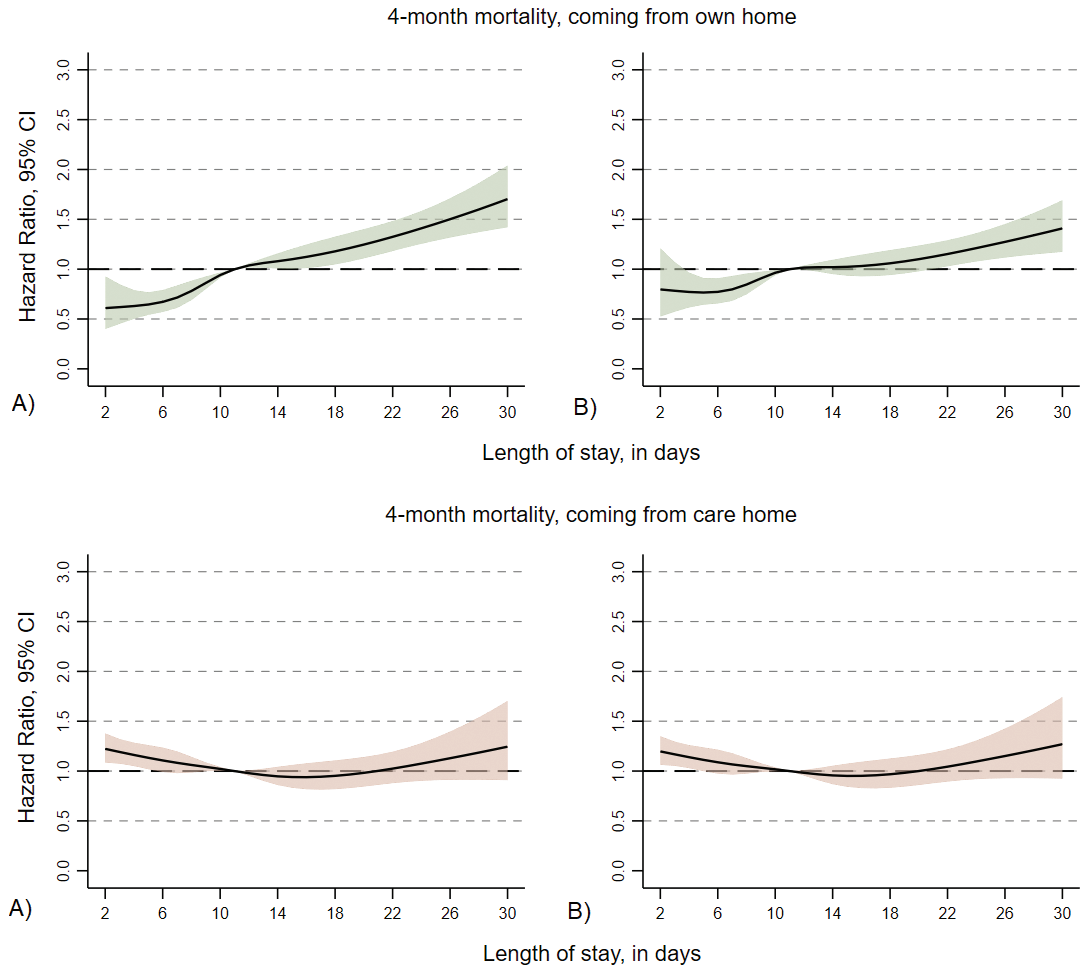
Hazard ratios for the association between days of LoS and 4-month mortality with 11 days as reference, stratified on living arrangements: (**A**) unadjusted model and (**B**) fully adjusted model (sex, age, ASA grade, and walking ability). LoS = length of hospital stay; ASA = American Society of Anesthesiologists physical status classification.

The interaction between LoS and living arrangement was statistically significant for all categories (*p* < .05; not shown). Table 3 and Figure 2 show the association between LoS and mortality stratified by living arrangement before hip fracture (ie, among people who came from their own homes, or those who came from a health care facility or care home). We found clear differences between the 2 subgroups. Among those who came from their own home, having a short LoS was proven protective for 4-month mortality; LoS 2–4 days HR 0.53 (95% CI: 0.38–0.74) and LoS 5–8 days HR 0.77 (95% CI: 0.67–0.89). The magnitude of the HRs marginally changed and remained stable in all models, with the fully adjusted model showing an HR of 0.65 (95% CI: 0.47–0.91) and 0.85 (95% CI: 0.74–0.98) for LoS 2–4 and 5–8, respectively. On the contrary, among those who came from either another health care facility or a care home, the shortest LoS was associated with a 17% increased risk of mortality, compared to the reference group (HR = 1.17, 95% CI: 1.05–1.30), while no statistically significant associations were found for any of the other LoS categories. None of the confounders, that were included in the models, altered the HRs considerably as seen in Table 3.

**Table 3.** Hazard Ratios for the Association Between LoS and 4-Month Mortality, Stratified by Type of Living Arrangement Before Admission

				Hazard Ratios, 95% CI			
	n	Cases	Absolute Risk, %	Model 1	Model 2	Model 3	Model 4
Own home (*n* = 34 290)							
LoS, in days							
2–4	1 786	37	2.1	**0.53 (0.38–0.74)**	**0.63 (0.45–0.88)**	**0.57 (0.41–0.79)**	**0.65 (0.47–0.91)**
5–8	6 960	289	4.2	**0.77 (0.67–0.89)**	**0.83 (0.72–0.95)**	**0.80 (0.70–0.92)**	**0.85 (0.74–0.98)**
9–12	10 953	729	6.7	Ref.	Ref.	Ref.	Ref.
13–23	12 695	1 084	8.5	**1.18 (1.07–1.29)**	1.08 (0.99–1.19)	**1.14 (1.04–1.26)**	1.07 (0.97–1.18)
24+	1 896	224	11.8	**1.63 (1.40–1.89)**	**1.42 (1.22–1.65)**	**1.54 (1.32–1.79)**	**1.38 (1.19–1.61)**
Health care facility or care home (*n* = 13 521)							
LoS, in days							
2–4	3 328	902	27.1	**1.17 (1.05–1.30)**	**1.19 (1.07–1.32)**	**1.14 (1.02–1.26)**	**1.16 (1.04–1.28)**
5–8	5 818	1 553	26.7	1.10 (0.99–1.21)	**1.11 (1.01–1.23)**	1.08 (0.97–1.18)	1.09 (0.99–1.19)
9–12	2 343	584	24.9	Ref.	Ref.	Ref.	Ref.
13–23	1 775	411	23.2	0.95 (0.83–1.08)	0.92 (0.83–1.05)	0.99 (0.87–1.12)	0.96 (0.85–1.09)
24+	257	66	25.7	1.11 (0.86–1.43)	1.07 (0.83–1.38)	1.19 (0.92–1.53)	1.14 (0.89–1.48)

*Notes:* LoS = length of hospital stay; CI = confidence interval; ASA = American Society of Anesthesiologists physical status classification. Model 1: adjusted for age and sex; Model 2: adjusted for sex and ASA score; Model 3: adjusted for sex and walking ability before the fracture; Model 4: adjusted for all factors above. Statistically significant hazard ratios are denoted in bold.

### Sensitivity Analyses

The stratified survival analyses for men and women and different age groups did not show any clear differences from the main analysis except for a tendency toward short LoS being more hazardous for those older than the age of 80 years (Supplementary Tables 1 and 2). The comparison of death rates between 13 and 30 days after admission for those who were at the hospital to those who had been discharged prior to that showed no significant difference, meaning that place of death did not influence the short-term death rates (not shown).

## Discussion

In this study, we show how the association between LoS and 4-month mortality after hip fracture is nonlinear with an increased risk of mortality for both short and long LoS and an “optimal” LoS around 11 days. This is in line with a previous Swedish study (7). However, we additionally show that when we adjust for patients’ health status, physical function, and living arrangements, a short LoS is no longer associated with an increased risk of mortality. In fact, when stratifying the analyses for the type of accommodation, those in independent living had a linear increase in the risk of death with increasing LoS, and the individuals who resided in care homes had a reversed association, although attenuated.

A long LoS was associated with mortality for both men and women at all ages. It is probable that other factors, such as complications during the hospital stay or unmeasured health issues, are both prolonging the hospital stay and increasing the risk of 4-month mortality for some individuals. This is supported by previous research showing that a short LoS decreases the risk of nosocomial infections (18) and, in turn, later adverse health outcomes. In our study, however, this association was attenuated and no longer significant for those individuals who came from a care home or any other health care facility. A possible explanation for this could be that care home residents are sent home earlier because health care is available at the care homes as well. However, the consequences of getting discharged early without the possibility of specialized care need to be further investigated in this group. In line with our results, van Dijk et al. (19) conclude that patients admitted from health care facilities or care homes are more vulnerable, have a higher risk of adverse outcomes, and need extra attention during the hospital stay and after discharge. However, as stated by Castelli et al. (20), the high death rate among hip fracture patients is probably not only due to the fracture itself but due to associated conditions and comorbidities that have put the individual at risk for a hip fracture in the first place. Consequently, the hip fracture might not be the cause of death but a symptom of frailty or vulnerability of a subgroup of older individuals who have high death rates. The hip fracture might in this case be the last tipping point that the frail individual is not able to recover from (21).

In the fully adjusted models, we saw that the covariates (ASA grade, walking ability, and living arrangements) were more important for the risk of mortality than LoS itself. This is in line with the results from Yoo et al. (10) who found both old age and high comorbidity load to be more strongly associated with mortality than LoS. These factors are related to poor health and are known to be associated with higher mortality in all patient groups (22). The fact that LoS does not matter as much for mortality as other known risk factors is reassuring and can be interpreted as, in general, Swedish hip fracture patients are staying in the hospital for as long as they need, and that the decision is made individually for each patient. The differences in cause of death between the LoS categories also imply that the different categories represent different health profiles. Still, the risk of being sent home too early remains for the most vulnerable. The higher frequency of “injuries/trauma” as the cause of death in the short LoS categories might indicate this. To be able to explore this further, future studies should not only focus on mortality but also hospital readmissions, changes in care utilization over time, and residual disability.

The strengths of this study include a large study sample that is collected from high-quality registers with close to full national coverage. In addition, the SHR contributes with more in-depth clinical details. Still, there are factors that might affect the association between LoS and mortality that this study did not consider. One limitation with this study is the possibility of confounding by indication. As mentioned above, it is possible that the patients stayed at the hospital for a long time just because they needed more care. By adding health-related and sociodemographic factors, we have tried to control for that, but it is likely that there is residual confounding. Examples of other factors that could have played a role in the association between LoS and mortality could be postsurgery complications, a specific measure of comorbidity and information about postsurgery mobilization and rehabilitation. Postsurgery complications such as infections, pulmonary emboli, and urinary tract infections could have affected the LoS and thus explain part of the observed association between LoS and mortality. However, the reported rate of any type of complications during the current hospitalization was low across the study population (less than 5%). In this study, we did not have access to information about the time of mobilization and rehabilitation proceedings; however, there are national guidelines for postfracture care including mobilizations and possibility of rehabilitation in Sweden (14). This means that the differences in mobilization and rehabilitation should mainly be due to health status, which is controlled for by ASA grade and walking ability. Another factor that could have been useful when interpreting the results is a specific measure of comorbidity. In this study, we chose to measure comorbidity with the proxy of ASA grade, a more clinical-friendly tool. In a recent publication, we showed that ASA grade and Charlson Comorbidity Index can be used interchangeably in this patient group (23). Last, in line with previous research (7), we started the time to event at discharge from the hospital, to make sure all individuals included in the study had an equal chance of staying as long as was intended for them. If those who died during the hospital stay had been left in the study, the associations with mortality and a short LoS might have been different. However, very few individuals were excluded due to death during the hospitalization (≈5%), so it is not likely that those individuals would alter the associations greatly. Because the management of hip fracture patients in other parts of the world might differ from this Swedish setting, where, for example, the rehabilitation of the patient is taken care of outside of the acute hospital, the results from this study might not be generalizable for all hip fracture patients worldwide. It is, however, likely that sociodemographic and health-related factors of the patients affect the association between LoS and mortality in other settings as well.

In conclusion, a long LoS in hospital after a hip fracture is associated with increased 4-month mortality risk even after taking patient characteristics into account. The association between mortality and a short LoS, however, is explained by hip fracture patients coming from care homes (and having higher mortality) being discharged early. Healthier fracture patients seem to benefit from a short LoS while the opposite is true for the frailer patients living in a care home. Overall, ASA score, type of accommodation, and walking ability prior to the fracture are more important predictors than the LoS for mortality after a hip fracture. This study highlights the importance of looking at different groups of hip fracture patients when exploring risk factors.

## Funding

This work was supported by the Kamprad Family Foundation for Entrepreneurship, Research and Charity (grant number 20190135). The funding source did not play an active role in the investigation.

## Conflict of Interest

The authors have no conflict of interest to declare.

## Supplementary Material

glab302_suppl_Supplementary_Tables
