# Supplementary material for: Hospital Length of Stay After Hip Fracture and It’s Association With 4-Month Mortality—Exploring the Role of Patient Characteristics
Source: J Gerontol A Biol Sci Med Sci. 2021 Oct 8;77(7):1472–7. doi: 10.1093/gerona/glab302 (PMC9255691; doi:10.1093/gerona/glab302)
Supplement: glab302_suppl_Supplementary_Tables [file glab302_suppl_supplementary_tables.pdf]

Supplementary Tables

Supplementary Table 1. Hazard ratios for the association between LoS and 4-month mortality, analyzed with Cox proportional hazards models (95% confidence intervals) and stratified by sex.

	Model 1	Model 2	Model 3	Model 4	Model 5
			**Women**		
**LoS**, in days					
2-4	**2.15 (1.93-2.39)**	**2.00 (1.79-2.22)**	**1.47 (1.31-1.63)**	1.00 (0.89-1.12)	1.00 (0.89-1.12)
5-8	**1.56 (1.43-1.71)**	**1.50 (1.38-1.65)**	**1.23 (1.12-1.35)**	0.9 (0.85-1.02)	0.93 (0.85-1.02)
9-12	Ref	Ref	Ref	Ref	Ref
13-23	0.97 (0.88-1.06)	0.91 (0.83-1.00)	0.99 (0.90-1.09)	**1.15 (1.04-1.26)**	1.07 (0.97-1.18)
24+	1.16 (0.98-1.38)	1.08 (0.91-1.28)	1.18 (0.99-1.41)	**1.44 (1.21-1.72)**	**1.30 (1.10-1.55)**
			**Men**		
**LoS**, in days					
2-4	**2.14 (1.87-2.46)**	**2.08 (1.81-2.38)**	**1.58 (1.38-1.82)**	1.13 (0.98-1.31)	**1.17 (1.01-1.35)**
5-8	**1.60 (1.42-1.79)**	**1.60 (1.42-1.79)**	**1.33 (1.18-1.50)**	1.04 (0.93-1.18)	1.09 (0.97-1.23)
9-12	Ref	Ref	Ref	Ref	Ref
13-23	1.00 (0.88-1.13)	0.95 (0.85-1.08)	1.05 (0.93-1.18)	**1.13 (1.00-1.28)**	1.11 (0.98-1.91)
24+	**1.47 (1.21-1.78)**	**1.31 (1.08-1.59)**	**1.48 (1.23-1.80)**	**1.72 (1.42-2.09)**	**1.58 (1.30-1.91)**

Model 1: controlled for age; **Model 2**: controlled for age and ASA grade; **Model 3**: controlled age and walking ability before the fracture; **Model 4**; controlled for age and living arrangements before admission; **Model 5**: controlled for all factors above

Supplementary Table 2. Hazard ratios for the association between categories of LoS and 4-month mortality, analyzed with Cox proportional hazards (HR, 95% significance level) and stratified by age groups.

	Model 1	Model 2	Model 3	Model 4	Model 5
			**65-79 years**		
**LoS**, in days					
2-4	**1.55 (1.23-1.94)**	**1.89 (1.50-2.38)**	**1.37 (1.09-1.72)**	0.88 (0.70-1.12)	1.06 (0.83-1.35)
5-8	1.12 (0.92-1.37)	**1.27 (1.03-1.55)**	1.10 (0.90-1.35)	0.83 (0.67-1.02)	0.95 (0.77-1.17)
9-12	Ref	Ref	Ref	Ref	Ref
13-23	**1.38 (1.13-1.69)**	1.22 (0.99-1.49)	**1.28 (1.05-1.56)**	**1.38 (1.13-1.69)**	**1.26 (1.03-)-1.54)**
24+	**1.80 (1.28-2.51)**	1.39 (0.99-1.94)	**1.58 (1.13-2.21)**	**1.86 (1.32-2.61)**	**1.51 (1.08-2.12)**
			**80+ years**		
**LoS**, in days					
2-4	**2.30 (2.10-2.52)**	**2.12 (1.94-2.32)**	**1.53 (1.39-1.67)**	1.02 (0.92-1.12)	1.03 (0.93-1.13)
5-8	**1.68 (1.56-1.81)**	**1.63 (1.51-1.76)**	**1.31 (1.21-1.42)**	0.98 (0.91-1.06)	0.99 (0.91-1.07)
9-12	Ref	Ref	Ref	Ref	Ref
13-23	0.95 (0.88-1.03)	0.90 (0.83-0.98)	0.98 (0.90-1.06)	**1.12 (1.03-1.22)**	1.07 (0.99-1.16)
24+	**1.24 (1.08-1.43)**	1.13 (0.98-1.30)	**1.26 (1.10-1.45)**	**1.53 (1.33-1.76)**	**1.40 (1.22-1.61)**

Model 1: controlled for sex; Model 2: controlled for sex and ASA grade; Model 3: controlled for sex and walking ability before the fracture; Model 4; controlled for sex and living arrangements before admission; Model 5: controlled for all factors above
